# Environmental impact of utilization of “produced water” from oil and gas operations in turfgrass systems

**DOI:** 10.1038/s41598-020-72069-5

**Published:** 2020-09-14

**Authors:** Sameera S. Shaikh, Mohammed H. Abu-Dieyeh, Fatima A. Al Naemi, Talaat Ahmed, Mohammad A. Al-Ghouti

**Affiliations:** Department of Biological and Environmental Sciences, College of Arts and Sciences, Qatar University, Doha, Qatar

**Keywords:** Pollution remediation, Environmental impact

## Abstract

This study attempted to use produced water (PW) to irrigate turfgrass species, *Cynodon dactylon* and *Paspalum *sp. Assessment on established grasses, heavy metal accumulation and germination tests for weeds and turf grass seeds were conducted to evaluate the impact of PW irrigation. *C. dactylon* depicted lower tolerance while *Paspalum *sp. showed better tolerance capacity towards PW. *C. dactylon* grown from seeds under greenhouse conditions were not able to tolerate more than 30% concentration of PW (4.5% salinity). In comparison to tap water irrigated turf grass, *Paspalum *sp. was found to accumulate higher concentrations of V and Pb in shoots and Cr, Ni and As in roots. The results of seed germination tests recommended that irrigation with PW is to be performed after turfgrass establishment. Germination tests also revealed that PW could encourage growth of the weed—*Chloris virgata* while it could discourage growth of *Amaranthus viridis* and *Launaea mucronata*. This study suggests that PW could be used for turfgrass as an alternative water resource but only after further research on the long-term scale.

## Introduction

In the Middle East, water consumption for irrigation is high and is attributed to the region's less fertile, arid lands. Concerning the State of Qatar, irrigation water requirements have almost doubled from 1990 to 2013, reaching close to 300 million m^3^ in the latter year^1^. Since, desalination of seawater is the major source of water for Qatar (contributing 57% of total volume for the year 2012)^1^, and a considerable amount of desalinated water is being used for irrigation purposes. Additionally, by 2050, Qatar is expected to have only 1–1,000 m^3^/capita/annum of freshwater resources^2^. Thus, to minimize the use of desalinated water for agricultural purposes and to ensure freshwater availability in Qatar, the use of alternative water resources, such as wastewater for agricultural purposes, seems ideal. The use of wastewater for irrigation is not a novel concept. Various types of wastewaters are being utilized to irrigate varied crops around the world^3–6^.

Given Qatar's Oil and Gas industry, one type of wastewater available in abundance in the country is the 'produced water'. It is a resultant waste product of the process of oil and gas extraction^7^. Produced water contains organic compounds, dissolved salts, suspended solid particles, emulsified oil and fracturing chemical compounds and additives such as corrosion inhibitors and biocide^7^. The suspended solids include asphaltenes, formation solids, scale and corrosion products, bacteria and waxes^7^. They also include metals and heavy metals in addition to petroleum hydrocarbons such as benzene, toluene, ethyl benzene and xylene (BTEX)^8,9^ and poly aromatic hydrocarbons (PAHs). Multiple inorganic salts are also present such as CaCl_2_, MgCl_2_ and NaCl and the salinity resulting from these may be as low as a few ppm to as high as 300 g/L^10^.

Produced water is of concern to environmentalists due to its aforementioned components and the large volumes of such components. The current disposal method involves injection of the produced water back into deep wells. This procedure may require high input of cost but may also be mandatory at certain oil production companies. Using produced water for irrigation provides an alternative to its disposal. Usage of produced water for irrigation is a new concept and thus, studies on the topic are recent and few in number. A new study in the U.S. employed produced water for irrigation in a similar fashion to this current study^11^. Given the high salinity and total organic content (TOC) levels of produced water, the authors tested salt tolerant non-food biofuel crops—Switch grass (*Panicum virgatum *L.) and Rapeseed (*Brassica napus *L.). They reported that the highest concentrations tested (salinity and TOC) significantly lowered the growth, health and physiological characteristics of both species. The authors concluded that the removal of organic matter to keep the TOC concentration less than 5 mg/L is required to maintain a sustainable biomass production rate^11^. Another study used produced water sourced from Wyoming and Montana in the U.S. on switch grass and corn, and four biofuel species—lemongrass, Japanese corn mint, common wormwood and spearmint. They concluded that prolonged use of produced water could have long-term deleterious effects on the soil and the plants, except if the produced water was treated or diluted with clean water^12^.

Treatment of produced water has been performed widely through the use of various physical and biological treatments^13^. However, in the past decades, common treatment methods for produced water have mainly consisted of membrane processes. These include forward osmosis (FO), nanofiltration (NF), ultrafiltration (UF) and microfiltration (MF)^13^. Prior treatment of the produced water before its use as an irrigation source provides a means to minimize the negative effects of the produced water's components on the irrigated crops.

However, due to risks associated with using produced water for growing food crops, non-food crops such as turfgrasses make a better candidate for wastewater treatment. Turfgrasses provide areas for leisure and sport activities and, more importantly, contribute to carbon sequestration. In addition, they are also used for land reclamation activities in areas that have been contaminated or areas that are industrial sites. Turfgrass covers very large areas around the world and the use of wastewater to irrigate turfgrasses has been conceptualized in the last decades and it has been applied in various parts of the world. For instance, in the state of Nevada in the U.S., more than 30 of the 53 golf courses utilize recycled water to irrigate greens, fairways and landscape plants^14^. A study in Portugal assessed the quality of turfgrass (*Cynodon dactylon*) as a response to varied irrigation regimes including wastewater^15^. They concluded that treated final effluents could be considered as an alternative to potable water for irrigation of golf courses. It has also been reported that *Cynodon dactylon* has a high ability to grow in high sodium containing—severely sodic—soils^16^. Hence, it is a potential candidate for receiving high salt containing produced water as an irrigation source. Bioaccumulation is a challenge posed by produced water usage. Bioaccumulation of components sourced from irrigation water is a common phenomenon and it hinders the application of wastewaters like produced water. The accumulation of heavy metals in soils due to irrigation sources has been reported in the Middle East and around the world^17–20^. Thus, understanding the bioaccumulation capacity of produced water and the deleterious effects it could bring about to the soil and to turf grasses is essential.

Weeds are a nuisance to fields of crops and to areas where turf grasses are grown, competing with the cultivated crop for nutrients, water and space. Weeds are known to have rapid growth and maturation^21^. A change in the nutrient availability, soil structure and the soil microbial community can induce a change in the weed community of the concerned area^22^. Since produced water contains organic matter and metals, the weed community structure is expected to change based on these parameters as they could lead to the enhancement of weed growth^22^. Hence, germination tests based on parameters such as effect of salinity, heavy metal concentration and hydrocarbons allow for the pre-understanding of weed dynamics.

Qatar's turfgrass area accounts for 701,628 m^2^ (Ministry of Municipality and Environment, Qatar, personal communication, 2017) which implicates the high water requirement for its maintenance. Statistical data has already proved the water insecurity of the country. Given that Qatar has an oil and gas based economy, we have an alternate source of water that is not only abundant but is also readily available—produced water. Although previous studies have evaluated and applied irrigation of turf grass with wastewater, a study testing produced water is novel and has been assessed in this study with three major objectives: Assessing turfgrass seed germination and turfgrass establishment and investigating seed germination of weed species as influenced by produced water irrigation.

## Methods

### Produced water samples

The produced water tested in this study was provided by TOTAL Qatar. The water was sourced from Al-Khalij offshore operation and transferred in a big container (1 m^3^) to be kept in storage outdoors, after the thorough consideration of all the safety issues that may take place due to any possible leaking. In each sampling, the salinity and pH were determined before any experimental work took place. Produced water (PW) samples were analyzed for different metals and other chemical constituents^23,24^. Table 1 shows TDS values, pH, chemical constituents, metal concentrations and other parameters for the (PW) samples.

**Table 1:** Chemical analysis of produced water in comparison with tap water. Adapted from^23,24^.

Properties and chemical constituents (mg/L)			Trace metal concentration (mg/L)			Hydrocarbon constituents and other parameters (mg/L) Parameter mg/L		
Parameter	Produced water	Tap water	Parameter	Produced water	Tap water	Parameter	Produced water	Tap water
EC (µS/cm)	240	0.1785	Li	4	0.011	Benzene	0.0395	ND
TDS	150,000–310,000	ND	B	38.6	ND	Toluene	0.0720	ND
TSS	6,760	ND	Ba	5.5	0.013	Ethyl benzene	0.0300	ND
pH	6.54	7.5	Bi	0.3390	ND	Xylene	0.0150	ND
F	4	ND	Al	0.1360	ND	Total diesel	0.1180	ND
Cl	122,000	5.1	As	0.0137	ND	Total PAHs	0.2925	ND
Br	710	< 0.1	Ag	0.0240	ND	TOC	2,430	ND
NO3	500	ND	Cd	0.0007	0.0002	BOD_5_	10	ND
PO4	4	ND	Co	0.0009	0.001	COD	8,983	ND
SO4	50	1	Cr	0.0111	0.001	Phenols	165.5	ND
CO3	134	ND	Cs	0.0240	ND	CN	0.50	ND
Na	61,000	3	Cu	0.0182	ND			
K	1,850	0.15	Fe	0.8414	ND			
Ca	10,700	39.2	Mn	0.2760	0.0075			
Mg	2,200	2.5	Pb	0.0525	0.009			
NH4	126	ND	Sr	750	0.021			
SAR (meq/L)	139.94	0.13	V	0.01	ND			
Ionic strength	3.79	0.00232	Zn	0.063	0.619			

*ND* not defined.

Prior to any assessment, the stock produced water samples were diluted according to the respective solution using tap water. The dilutions were prepared frequently and stored for short periods.

Given the safety issues of directly applying produced water to a land, experiments were set up using pots both inside and outside the greenhouse. Based on preliminary results, the subsequent experiments considered lower produced water dilutions and salinity as a factor. In addition, a further experiment was set up outdoors to simulate natural conditions. Germination tests of weed seeds were conducted to understand the effect of produced water on weed dynamics.

### Assessing the effect of different concentrations of produced water on turf grass (*Cynodon dactylon*) establishment

2 g of turfgrass seeds, *Cynodon dactylon* (Cȇsped gobi, Semillas fito, Spain) were sown in 30 pots (20 cm diameter) containing 60% sandy loam soil and 40% Peat moss soil. The pots were left for a period of 2 months to allow the grass to be established prior to treatment. The experiment included one factor with a completely randomized design (CRD) and it was set up inside a greenhouse (24 ± 2 °C with 15 h of light/day at photon flux density minimum of 350 ± 50 µmol m^−2^ s^−1^). Six replicates were designated for each treatment and the pots were irrigated once a week with 200 mL of the assigned treatment. Treatment volume was determined based on irrigation regimes during the establishment period. The treatment levels were—0% (tap water), 25% produced water, 50% produced water, 75% produced water and 100% produced water concentrations. The dilutions were prepared using tap water. The dilutions were prepared frequently and stored for short periods in the refrigerator. Two weeks post treatment, the irrigation was alternated with tap water to de-stress the turf system and reduce the accumulative effects of heavy metals, salinity and other produced water components.

The percentage of green biomass was observed and estimated every week using a scale rank of 0–100%, 0% for no green grass in the pot, and 100% for complete green and healthy grass in the whole pot and a percentage of green grass compared to control treatments for other results. After a period of 14 weeks, the green biomass for each pot was collected and put into a labeled paper bag. The root system of each pot was carefully cleaned from soil, washed with tap water and then put in a separate paper bag. All paper bags were placed inside an oven for 3–4 days 80 °C. The dry weight of the above ground biomass and the below ground root biomass were determined for each pot.

### Assessing the effect of different produced water and saline water concentrations on turf grass (*Cynodon dactylon*) establishment

Based on the results obtained from the previous experiment (i.e. see “Assessing the effect of different concentrations of produced water on turf grass (*Cynodondactylon*) establishment” section), lower concentrations of produced water were tested in order to reach the proper dilution of produced water needed to maintain healthy grass. In addition, different concentrations of saline water treatments were also added in order to determine if the negative effect was due to the salinity of the produced water. 2 g of turf grass seeds *Cynodon dactylon* (Cȇsped gobi, Semillas fito, Spain) were sown in 44 pots (20 cm diameter) containing a mixture of 60% sandy loam soil and 40% peat moss soil. The pots were left for a period of 2 months to let grass established prior to treatment. The experiment was one factor and four replications with a completely randomized design (CRD) and set up inside a greenhouse at similar conditions like experiment (see “Assessing the effect of different concentrations of produced water on turf grass (*Cynodondactylon*) establishment” section). The treatment levels were: 0% (tap water), 10% produced water, 20% produced water, 30% produced water, 40% produced water and 50% produced water.

The saline water concentrations were matched to the produced water concentrations. The calculations were made using 100% salinity as 150 g/L (average of total measured salinity of produced water). The saline water concentrations tested were—15 g/L saline solution (designated as 1.5% S), 30 g/L saline solution (designated as 3% S), 45 g/L saline solution (designated as 4.5% S), 60 g/L (designated as 6% S) and 75 g/L (designated as 7.5% S). The irrigation was accomplished using 200 mL per pot of the assigned treatment, once a week. Two weeks post treatment, the irrigation was alternated with tap water to de-stress the turf system.

The percentage of green biomass was visually estimated every week using a scale rank of 0–100%. 0% stood for no green grass in the pot, 100% for complete green and healthy grass in the whole pot, and a % in between of green grass when compared to the control treatments for the other results. After a period of 14 weeks, the green biomass for each pot was collected and put into a labeled paper bag. The root system of each pot was carefully cleaned from the soil, washed with tap water and then put into a separate paper bag. All paper bags were placed inside an oven for 4 days at 80 °C. The dry weight of the aboveground biomass and the belowground root biomass were thus determined for each pot.

### Assessing the effect of different produced water concentrations and saline water concentrations on established turf grass (*Paspalum* sp.) on a larger scale and outdoor conditions

Ready turf grass rolls of *Paspalum* sp*.* which are commonly used in Qatari turfgrass systems were obtained upon request from the Ministry of Environment, Public parks department. 21 large pots of the size—65 cm × 25 cm × 20 cm were procured. Although they had drainage systems, the pots were designed in a way to prevent water leakage and thus had no outlets. Seven treatment levels were designated—0% (tap water), 10% produced water, 20% produced water, 30% produced water, 15 g/L saline solution (designated as 1.5% S), 30 g/L saline solution (designated as 3% S) and 45 g/L saline solution (designated as 4.5% S). Three replications were assigned for each treatment. The experiment followed completely randomized design (CRD) with three replications and was set up outdoors to simulate natural conditions.

The pots were irrigated twice weekly with 250 mL/pot of assigned treatment each time. This treatment volume and schedule was determined based on water requirements of the grass observed during the establishment period. The irrigation was alternated with tap water (in the third week) to de-stress the turf system. The percentage of green biomass was visually estimated biweekly using the aforementioned method. After a period of 10 weeks, the green biomass for each pot was collected and put into a labeled paper bag. The root system of each pot was carefully cleaned from soil, washed with tap water and then put into a separate paper bag. All paper bags were placed inside an oven for 4 days at 80 °C. The dry weight of the aboveground biomass and the belowground root biomass were thus determined for each pot.

### Metal digestion and ICP analysis

Shoot and root samples from treatments—0%, 10%, 20% and 30% PW were collected at the end of the previous experiment (i.e. Assessing the effect of different produced water concentrations and saline water concentrations on established turf grass (*Paspalum* sp.) on a larger scale and outdoor conditions). The samples were dried at 100 °C overnight after which they were manually ground to a powder form. 0.25 g of the sample was weighed and added to heat resistant tubes. To this, 5 mL of concentrated nitric acid was added. SRM 15151—apple leaves were used as reference material. One sample (10% PW roots) was duplicated to ensure a validation of the measurements. A spike was prepared by taking 0.1 mL of standard 100 ppm (ICUS-2959) and diluting it to 100 ppb. Two blanks were also prepared containing the acid only. All samples were capped and placed on a hot block set at 105 °C. After 2 h, the sample tubes were uncapped and the hot block temperature was increased to 130 °C to allow for evaporation. Following evaporation, the residue was mixed with 3 mL of conc. nitric acid and 1 mL of hydrogen peroxide and the sample tubes were allowed to boil at 155 °C in the hot block. The samples were then transferred to measuring flasks and their volume was completed to 50 mL using distilled water. The digested samples were then filtered twice using 0.25 µm filters to remove the precipitates. The filtered samples were then analyzed for metals—vanadium, chromium, manganese, nickel, cobalt, zinc, arsenic, cadmium and lead through ICP Analysis.

### Seed germination experiments

Samples of produced water analysis showed that it contained—nickel 3.2 ppb, zinc 49.7 ppb, cobalt 0.75 ppb, lead 48.86 ppb (Table 1) in addition to a high salinity of 150 g/L. Therefore, germination tests on turf grass seeds (*Cynodon dactylon*) and weed species that are known to be associated with turf grass systems were performed using concentrations of the above mentioned heavy metals in addition to produced water and saline water treatments. Chloride salts of each of the metals was used to prepare the following concentrations nickel 3.2 ppb, zinc 49.7 ppb, cobalt 0.75 ppb, lead 48.86 ppb.

#### Germination test of turf grass (*Cynodon dactylon*) seeds

Seeds of turfgrass, *Cynodon dactylon* (Cȇsped gobi, *Cynodon dactylon,* Semillas fito, Spain) were surface sterilized using 5% sodium hypochlorite solution for 2 min and were followed by a washing with distilled water 3 times. 10 seeds of turfgrass (*Cynodon dactylon*) were placed in a Petri dish layered with a cheesecloth that was priorly soaked with 3 mL of the assigned treatment solution and then sealed with parafilm. 4 replicates were prepared for each treatment. The treatments tested were—0% control (distilled water), 1% produced water, 5% produced water, 10% produced water, 20% produced water, 1.5 g/L saline water (designated as 0.15% S), 7.5 g/L saline water (designated as 0.75% S), 15 g/L saline solution (designated as 1.5% S) and 30 g/L saline solution (designated as 3% S), nickel chloride 3.2 ppb, zinc chloride 49.7 ppb, cobalt chloride 0.75 ppb and lead chloride 48.86 ppb. The Petri dishes were placed in a growth chamber at 28 °C for 14 days. Distilled water was added (~ 2 mL) upon requirement to prevent dry out. The plates were observed daily for seed germination. The appearance of the white radicle was used as an indicator of germination. The germinated seeds were counted on a daily basis for 14 days. Accumulative of percent germination was documented for every treatment in all the experiments. The whole experiment was repeated once with statistically similar results.

#### Germination test of seeds of weed species associated with turf grass

Seeds of 6 common turf grass weeds were collected from turf grass fields inside Qatar University’s campus. The seeds were cleaned from husk and other residues, and then kept in labeled paper bags in a refrigerator. Seed germinability tests revealed that seeds of 3 out of 6 species were not able to germinate. The tested species were *Amaranthus viridis, Launea mucronata, *and* Chloris virgata.* The seeds were soaked in 5% sodium hypochlorite solution for 2 min and were followed by a washing with distilled water 3 times. 10 seeds were placed in a Petri dish layered with a cheesecloth that was priorly soaked with 3 mL of the assigned treatment solution and then sealed with parafilm. Four replicates were prepared for each treatment. The treatments tested were the same as in above-mentioned experiment (i.e. Germination test of turf grass (*Cynodon dactylon*) seeds). The Petri dishes were placed in a growth chamber at 28 °C for 14 days. Distilled water was added (~ 2 mL) upon requirement to prevent dry out. The plates were observed daily for seed germination. The appearance of the white radicle was used as an indicator of germination. The germinated seeds were counted on a daily basis for 14 days. Accumulative of percent germination was documented for every treatment in all experiments. During the experiment seeds of *Malva neglecta, Launaea capitata* and *Oligomeris subulata* were found to be non-viable and the experiment was then continued with *Amaranthus viridis*, *L. mucronata* and *Chloris virgata.*

### Data analysis

One-way ANOVA at *P* ≤ 0.05 was used to test the significance among treatments and the significance in each measured parameter. Tukey’s test was used for mean comparisons at *P* ≤ 0.05. SigmaStat 4.0 was used to perform the data analysis. Data from the two experimental trials of turfgrass seed germination experiment were subjected to the Bartlett test for the homogeneity of variances. Data for all measured variables were homogeneous and the two experiments were pooled and analyzed as one with 8 replications.

## Results and discussion

### Effect of produced water on turfgrass

**Figure 1:**
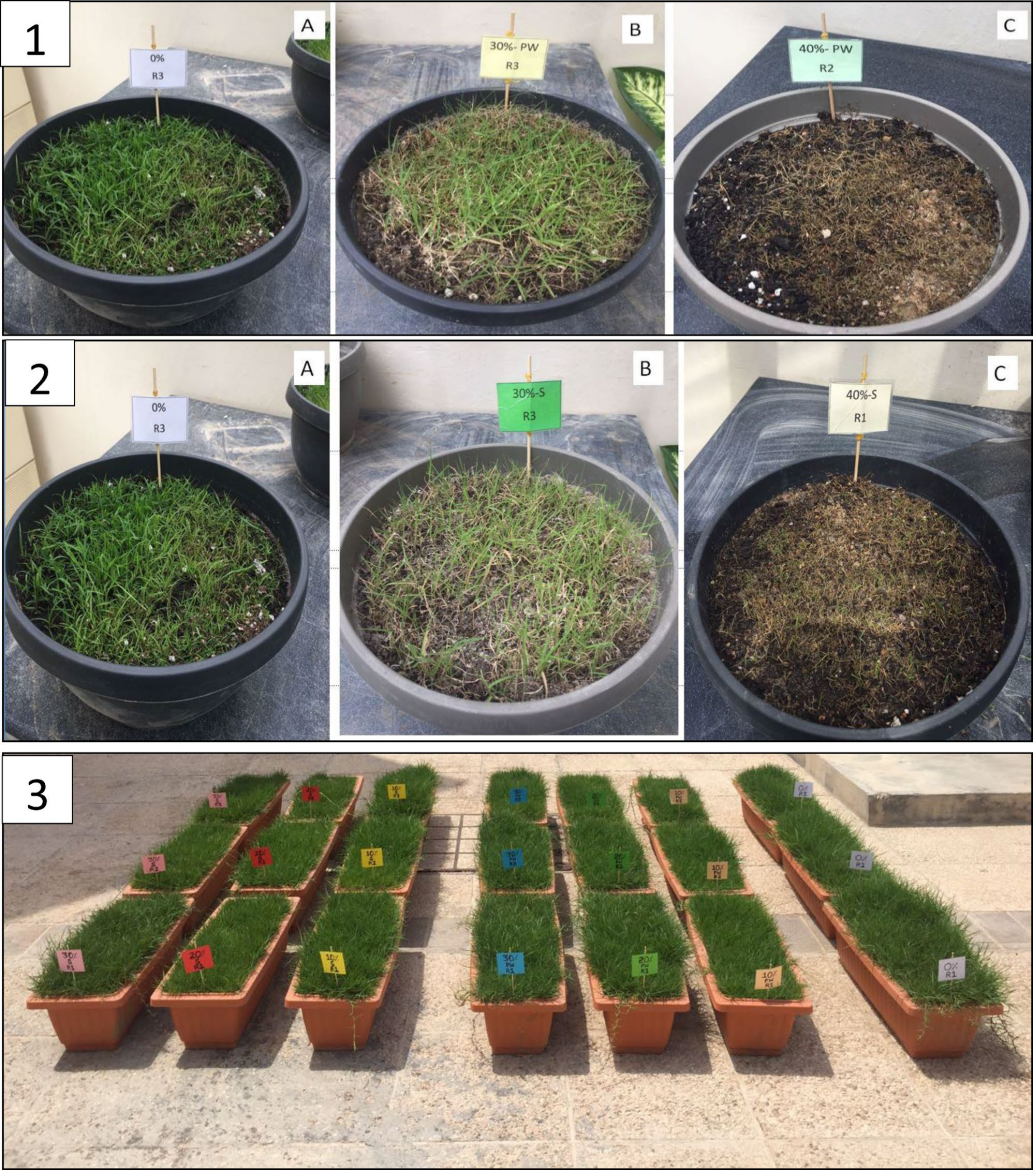
Photos for the effect of different produced water (PW) and salinity treatment on turfgrass growth after being subjected to 14 weeks of irrigation regimes. (**1**) Upper pictures: Effect of different concentrations of produced water on turfgrass (*Cynodon dactylon*), Tap water [A], 30% PW [B] and 40% PW [C]. (**2**) Middle pictures: Effect of different concentrations of salinity on turfgrass (*Cynodon dactylon*), 0% (tap water) [A], 4.5% NaCl (shown as 30% S) [B], and 6% NaCl (shown as 40% S) [C], (**3**) R-L Effect of different concentrations of produced water (0–30%) and salinity (0–4.5% NaCl) on turfgrass *Paspalum* sp.

**Figure 2:**
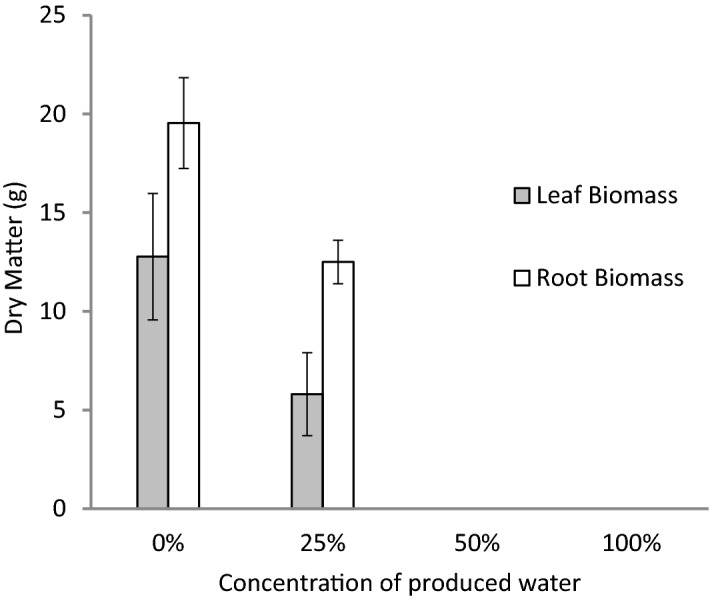
Effect of concentrations of produced water (0% refers to tap water) up to 100% produced water on biomass (dry matter) of above and belowground biomass of turfgrass (*Cynodon dactylon*) after being subjected to 14 weeks irrigation regimes. The grass was grown in 20 cm pots and placed under greenhouse conditions. Error bars represent the standard error of the means (*n* = 6).

**Figure 3:**
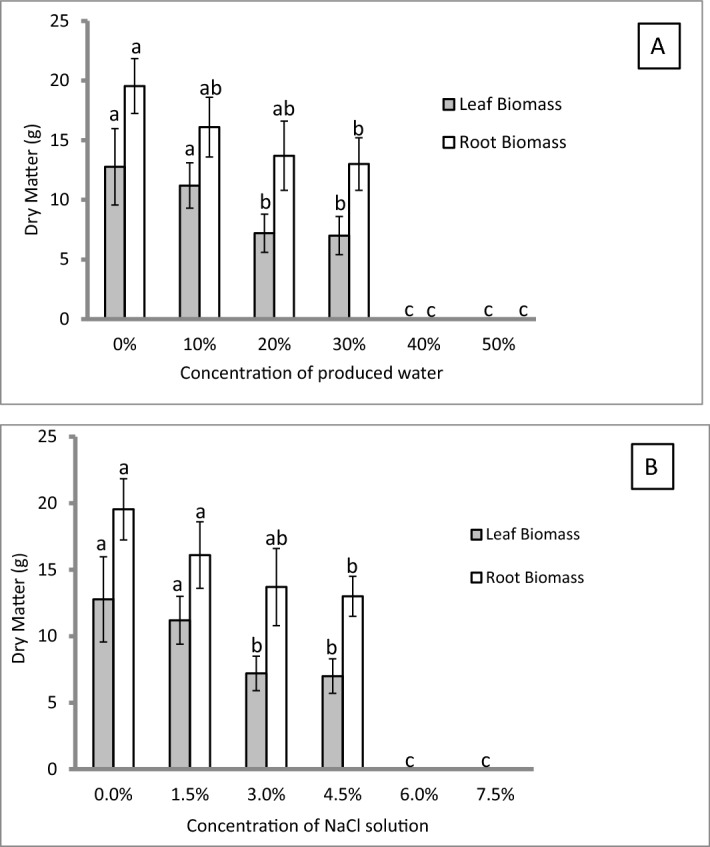
Effect of concentrations of produced water (**A**) and saline water concentrations (**B**) on turf grass (*Cynodon dactylon*) biomass after being subjected to 14-weeks irrigation regimes. The grass was grown in 20 cm pots and placed under greenhouse conditions. Error bars refers to standard error of the means. Within leaf or root biomass, any common letter between treatments refers to no significance at *P* ≤ 0.05 using Tukey’s test (*n* = 4).

Second week post produced water treatment, *Cynodon dactylon* grass started to dry without future recovery. Only pots irrigated with 25% produced water concentration were tolerant and survived up to the end of experiment (14 weeks). Although the grass was able to survive at 25% PW irrigation (Fig. 1), the green biomass and the dry matter both above and below the ground were significantly reduced (Fig. 2) compared to the turf grass biomasses subjected to tap water irrigation. The decline in grass biomass might be due to salinity factors or other toxic substances available in the raw produced water. Therefore, the experiment was repeated under similar conditions however this time salinity treatments were added. Results obtained were almost similar to the results of the first experiment. Above 30% PW irrigation killed the grass (Fig. 1-1). Interestingly, salinity treatment of 4.5% (equivalence to salinity in 30% PW) showed similar grass biomass like the 30% PW treatment (Fig. 1-2). Almost similar trends were obtained on coverage percentage and again the salinity treatment effects almost matched the effects obtained from produced water treatments. After 14 weeks of irrigation, results from dry matter biomass, indicated a statistically significant (*p* ≤ 0.05) reduction in leaf dry weight starting from 20% PW and the matching 3% salinity treatments, while a significant reduction (*p* ≤ 0.05) in root biomass started from 30% PW or the matching equivalence of salinity treatment (4.5%) was observed (Fig. 3).

**Figure 4:**
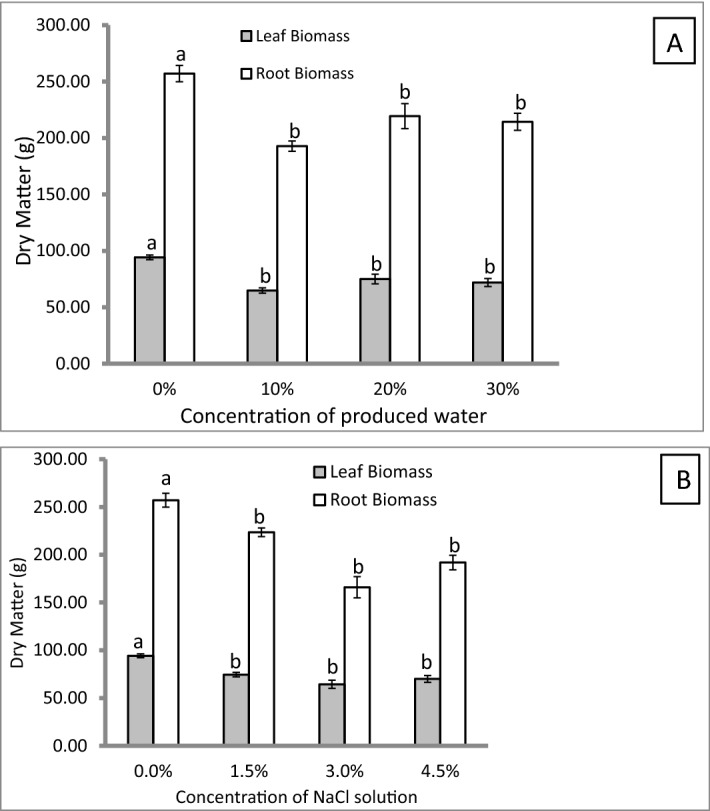
Effect of concentrations of produced water (**A**) and saline water concentrations (**B**) on turf grass (*Paspalum* sp.) biomass (%) after being subjected to 10 weeks irrigation regimes. The grass was grown in 65 cm × 25 cm × 20 cm under outdoor conditions. Error bars refers to standard error of the means. Within leaf or root biomass, any common letter between treatments refers to no significance at *P* ≤ 0.05 using Tukey’s test (*n* = 3).

Furthermore, another experiment was established in outdoor conditions using the turf grass species *Paspalum* sp., which is commonly used in Qatari turfgrass systems. The grass employed in the experiment was planted from rolls of seedlings and under outdoor conditions. In this experiment, we aimed to simulate the natural conditions of turf grass growth in Qatar. The obtained results indicated a better tolerance of *Paspalum* sp. to the produced water and salinity treatment. Figure 1-3 shows the appearance of the experimental turfgrass under all of the treatments following 10 weeks of irrigation. It is clear that there are neither negative effects on the green biomass due to the produced water nor due to the saline irrigation. However, Fig. 4 shows a statistically significant reduction (*p* ≤ 0.05) of dry weight of above and belowground parts under both treatments. Experimentation on *C. dactylon* and the similarity in the results obtained in produced water treatments and salinity treatments was an indication that negative effects on the treated grass could be primarily due to NaCl concentration in the raw produced water.

Based on obtained results, *C. dactylon* can tolerate up to 30% PW treatment but with loss in both aboveground biomass coverage% and biomass (leaf and root) while *Paspalum* sp. can at least tolerate 30% PW treatment with no loss in aboveground biomass coverage% but with reduction in biomass (leaf and root dry weight). A decrease in biomass of plants treated with produced water was an expected scenario. A change in irrigation regime is bound to bring about significant changes in the biomass of treated plants. Interestingly, similar biomass reduction results have been reported in studies that also tested the utilization of produced water for irrigation of varied plants^12,13^. It has been reported that, in general, turf grass species are efficient in utilizing nutrients from sources like wastewater^25^. The differences in growth patterns observed in *C. dactylon* and *Paspalum* sp. of the current study suggest that *Paspalum* sp. may have higher efficiency in utilizing the nutrient available in produced water as compared to *C. dactylon.* Different species have different tolerance capacities and have different growth requirements. The experimental conditions might also play a role in growth abilities of the turfgrasses. A study reported that difference in experimental conditions might relieve or worsen stress that is induced by salinity^26^. Since, the experiment concerned with *C. dactylon* was an indoor/greenhouse experiment and that concerned with *Paspalum* sp. was an outdoor experiment simulating natural conditions, differences in their response to produced water and its salinity was likely to occur. In addition, *Paspalum* sp. may have an inherent capacity of higher tolerance. It has been reported that *Paspalum* sp. such as *Paspalum vaginatum* have high salinity tolerance^27^ and this can be an explanation for its ability to better withstand both produced water and saline water concentrations in comparison to *C. dactylon.* The higher salt tolerance of *Paspalum *sp. may originate from the possession of 'up-regulated' stress defensive proteins that are unique to them^28^. Hence, the results obtained indicate that in the application of produced water for irrigation in Qatar, *Paspalum* sp. could be the better choice. This is advantageous since *Paspalum* sp. happens to be the most commonly used turfgrass system in Qatar.

### Metal digestion and ICP analysis

**Figure 5:**
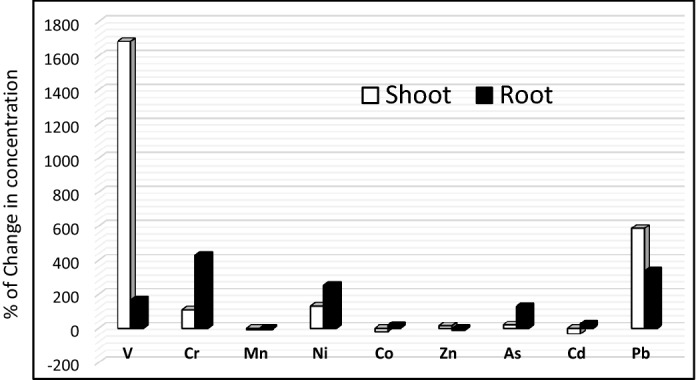
Change in concentration of heavy metals in the below (root) and aboveground (shoot) parts of turf grass after 10 weeks of irrigation regime using diluted produced water.

Preliminary investigation of the fate of heavy metals in the plant parts after being subjected to produced water irrigation was performed and results are shown in Table 2 for aboveground biomass and roots. According to the results of the ICP analysis, some elements were mainly accumulated in shoots i.e. V and Pb and others were more accumulated in the roots i.e. Cr, Ni, and As (Fig. 5). Metal digestion and ICP analysis of shoots and roots of *Paspalum* sp. treated with 10% PW–30% PW depicted accumulation of certain metals in shoots while others accumulated in roots (Fig. 5). Vanadium (V) and lead (Pb) were found to accumulate in the shoots of 10% PW–30% PW in higher concentration as compared to 0% treatment. It was reported that in mine tailings having high concentrations of Pb, *Paspalum *sp. was found to naturally colonize the region, thus asserting its ability to tolerate high Pb concentration and its possible usage in revegetation of Pb tailings^29^. In contrast, chromium (Cr), nickel (Ni) and arsenic (As) accumulated in the roots in higher quantities compared to the control (0%) treatments. This is supported by the fact that *Paspalum *sp*.* such as *Paspalum racemosum*, and *Paspalum tuberosum* are known to be hyper accumulators of arsenic (As)^30^. Interestingly, the 10–30% PW treated grass also lost certain metals, making their concentrations lower than the concentrations observed in the 0% tap water treatment. Their shoots had lowered concentration of manganese (Mn) cobalt (Co), and cadmium (Cd), while their roots had lowered concentration of manganese (Mn) and zinc (Zn). Our results are similar to the paper^31^ that studied Ni accumulation in *C. dactylon* and to that also studied element accumulation in *C. dactylon* which was irrigated with produced water^32^. Another study also reported similar accumulation of metals including Pb, Cd and Ni in parts of plants that were irrigated with wastewater^33^. Due to their long-term persistence in nature, heavy metals are an issue of concern. Bioaccumulation of heavy metals in turfgrass may provide a way for heavy metal removal from produced water and avoid heavy metal accumulation in soils. Phytoextraction, a strategy of phytoremediation allows heavy metal removal by their uptake through plant's shoots and roots, followed by removal of the plant from the site^34^. A longer study would completely ascertain the ability of *Paspalum *sp. to bio-accumulate heavy metals of produced water and act as a phytoextractor.

**Table 2:** ICP analysis of shoots and roots of turf grass *Paspalum *sp. treated with tap water (control) or different concentrations of produced water (PW).

Metal	Concentration in shoot tissues (mg/kg)				Concentrations in root tissues (mg/kg)			
	Control	10% PW	20% PW	30% PW	Control	10% PW	20% PW	30% PW
V	0.582	11.55	11.9	7.734	27.81	70.5	86.79	67.07
Cr	27.89	100.5	21.12	54.17	23.57	151.4	112.8	111.4
Mn	598.8	476.2	700.1	490.8	694.3	552.1	805.9	579.9
Ni	16.37	52.27	32.5	28.78	22.21	91.91	74.84	68.84
Co	5.052	4.188	4.694	3.485	18.99	17.49	33	17.05
Zn	356.6	305.1	447.5	477.2	445.2	376.8	352.3	464.8
As	3.1	3.958	3.498	ND	7.459	18.81	22.34	9.911
Cd	0.414	0.315	0.31	0.227	0.677	0.807	1.128	0.657
Pb	4.363	30.99	25.6	33.35	12.55	43.63	66.84	54.47

*ND* not defined.

### Effect on germination of turf grass seeds

**Figure 6:**
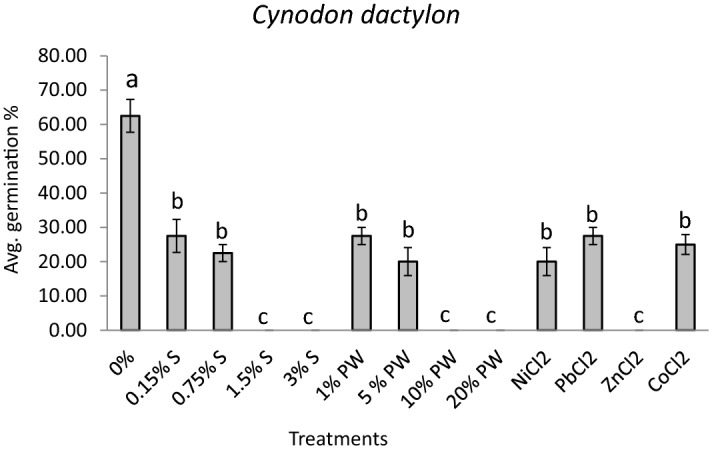
Average percentage of germination (8 replicates with 10 seeds/replicate) at 14 days for *Cynodon dactylon* subjected to varied treatments. Error bars refers to standard error of the means. Any common letter between treatments refers to no significance at *P* ≤ 0.05 using Tukey’s test.

Seed germination experiments were completed to investigate the effect of heavy metals found in produced water on the germination of turfgrass seeds. All kinds of heavy metals significantly decreased (*p* ≤ 0.05) the germination potential of the seeds of *Cynodon dactylon* turfgrass compared to control (Fig. 6), and hence, it can be concluded that the seeds were affected by both, the presence of NaCl and the presence of tested metals, in the produced water. Nickel is known to be toxic to plants even in low quantities^35^. Lead is known to be a phytotoxin^36^. Cadmium is also reported to be phytotoxic, while zinc has been reported to inhibit germination in certain species^37^ and thus could have inhibited the germination capacity of *C. dactylon*. This data suggests that while already established *C. dactylon* may tolerate up to 30% PW concentrations, germinating *C. dactylon* using produced water is not recommended. Irrigation of *C. dactylon* with produced water should be performed after it is well established. However, removal of salinity and of metals through produced water treatments may enhance the germination capacity of *C. dactylon* seeds.

### Effect on germination of weed seeds

**Figure 7:**
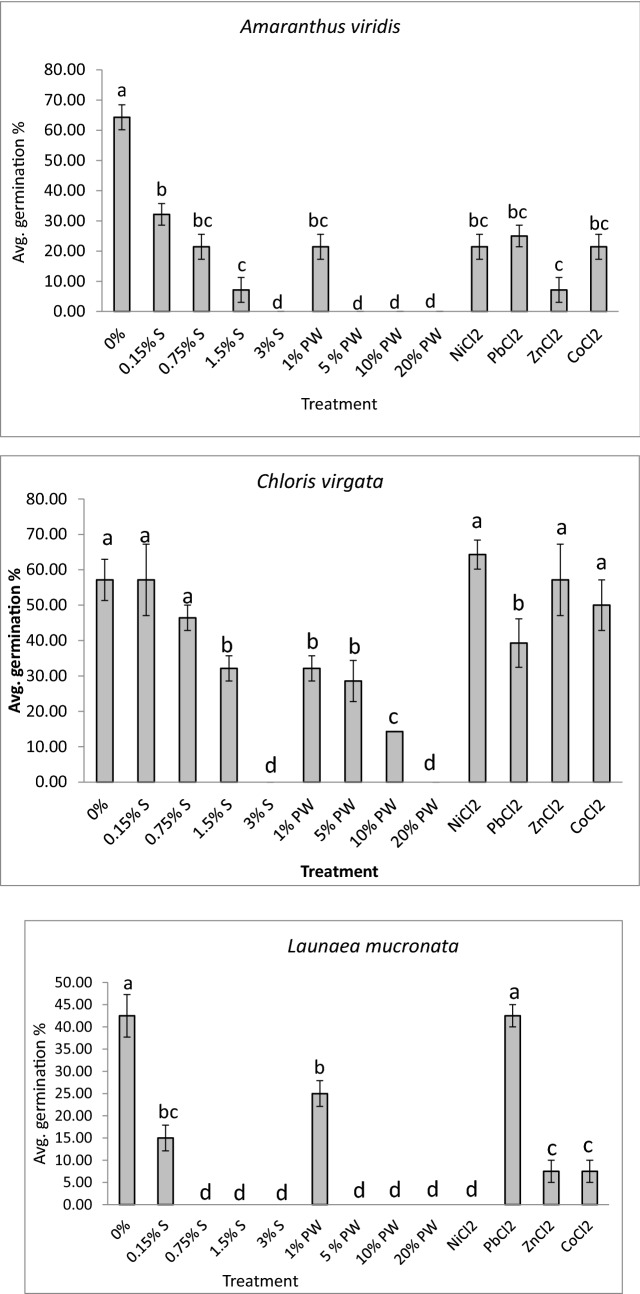
Average percentage of germination (4 replications with 10 seeds/replicate) for three weed species that are common to Qatari turfgrass after being subjected to varied produced water (PW), salinity (S) and heavy metal treatments. Error bars refers to standard error of the means. Any common letter between treatments refers to no significance at *P* ≤ 0.05 using Tukey’s test.

Seeds of weeds encountered in the turf grass were collected to be assessed for germination under different heavy metal treatments. Different weeds showed different germination potentials after being treated with different heavy metals (Fig. 7). For example, the most common weed species grown in turf grass systems of Qatar is *Amaranthus viridis.* The germination of the species was significantly reduced under all treatments (PW, salinity and heavy metals). Weeds are capable of disrupting vital ecosystem processes and out compete native species. Having a successful germination is extremely crucial in the life cycle of seeds^38^. In modified environmental conditions, seeds that can modify their germination behavior are highly likely to survive and establish themselves^38^. Thus, the germination capacity of weed seeds gives an indication of its survival rate when subjected to produced water irrigation.

The weed species *Amaranthus viridis* was discovered to be tolerant of salinity between 0.15–1.5% S but with lowered germination capacity (Fig. 7). However, the seeds could not germinate in produced water concentrations higher than 1% the stock PW solution suggesting that factors other than salinity affect its germination. Metal treatments also reduced germination capacity of *A. viridis*. Based on the results, it can be assumed that fields that are irrigated with produced water would discourage the growth of *Amaranthus viridis,* thus decreasing competition between turfgrass and weed species. This is advantageous as *Amaranthus viridis* is an invasive species characterized by rapid growth with high seed production rate^39^. It is known to grow well in very sandy soils and heavy organic soils^40^, and hence, could have been a nuisance if its growth was encouraged by produced water irrigation. In addition, it would lower costs for its removal and management as a result of the combination of hand weeding, fallow land management, and pre-post emergence herbicide that is required to control unwanted *A. viridis* growth^40^.

On the contrary, the weed *Chloris virgata* was observed to germinate with no significant differences between 0% treatment, 0.15% S, 0.75% S, NiCl_2_, ZnCl_2_ and CoCl_2_ treatments. 10% PW lowered germination percentage as compared to all treatments (Fig. 7). No germination was observed in 3% Salinity and 20% PW treatments. It can be suggested that *C. virgata* was affected primarily by a salinity higher than 1.5% since the germination of seeds was reported in metal treatments. It can be concluded that *C. virgata* weed can germinate and grow in fields irrigated with produced water if the concentration used is below 20% PW for a duration of 14 weeks. Concentration of produced water higher than 20% PW may discourage their growth but it would also deter growth of the turf grass. *Chloris virgata* is recognized as a halophyte species commonly growing in saline areas and degenerated grasslands^38^. It follows a C4 photosynthetic pathway giving it ability to grow in desert conditions and be drought resistant^41^, Hence its ability to germinate in both produced water and saline water. Thus, produced water concentration used for irrigation of turfgrass needs to be chosen in a manner to maximize turfgrass growth and to minimize the growth of *C. virgata.*

Seeds of *Launaea mucronata* could not germinate in 0.75% S–3% S, 5% PW–20% PW and NiCl_2_ treatments (Fig. 7). Interestingly, seeds germinated well in PbCl_2_ solution with no significant differences as compared to 0% treatment while 0.15% S, 1% PW, and ZnCl_2_ and CoCl_2_ treatments significantly decreased germination in comparison to the 0% treatment. It can be said that fields that have been irrigated with produced water would have reduced growth of *Launaea mucronata* and hence decreasing competition between turf grass and the weed species. It would also lower costs for the weed’s removal and management.

Change in weed dynamics due to irrigation sources is commonly observed. For instance, it was reported that the use of yeast wastewater exerted impact on weed communities^22^. When it’s compared to a freshwater irrigated and rain fed site, the yeast wastewater irrigated site had significantly higher vegetation cover and increased species' frequency, dispersion, richness and density. The vegetation cover obtained with yeast wastewater was twice to that of fresh water. The authors thus reported that the irrigation source has the ability to affect the relative abundance and the species composition of the weeds^22^. Similarly, produced water can also affect weed composition and abundance as observed in the results provided above, by encouraging growth of some species while discouraging that of others. Hence, thorough evaluation of the effects that produced water would have on turfgrass associated weeds is critical.

## Conclusions

The two tested turf grass species depicted a varied degree of tolerance and growth ability. *C. dactylon* was reported to be able to withstand up to 30% PW maximum concentration. The incorporation of a salinity factor in the experiment gave insight into understanding that NaCl concentrations could be the primary cause of the observed effects on *C. dactylon* turfgrass and hence the treatment for salt removal can allow higher concentrations of produced water to be used. The experiments conducted on *Paspalum* sp. suggested that it has a much higher capacity to tolerate salinity as well as produced water as a whole. It can withstand at least 30% PW/4.5% salinity. If long term experiments, that take into account the accumulation of salt and metals in the soil, were to be conducted to confirm these results, *Paspalum* sp. could potentially be used in areas in Qatar that are to be irrigated with produced water. However, as mentioned, the grass requires that it should be well established prior to the treatment in order to maximize its growth. In addition, a study conducted over a period of two seasons would further allow the understanding of the ability of the studied turf grass species to withstand produced water treatments.

Accumulation of metals in *Paspalum *sp. could indicate its ability to be used as means of metal removal or to be utilized in bioremediation projects. Use of produced water is expected to discourage growth of *Amaranthus viridis* and *Launaea mucronata* but may have no effect on the growth and abundance of *Chloris virgata.* More species of weeds need to be analyzed for their response to produced water. This will allow for the development of proper weed management strategies and precise management of cost. The concentration of the produced water chosen for irrigation, hence, is the key determinant on the effect it could have on the growth of the turfgrass and the growth and abundance of weeds.. In conclusion, if confirmed in the long term, produced water could be a viable, alternative irrigation source that could be used for irrigating turf grass. That is, if the suitable turf species are chosen, the area-requiring irrigation is well studied, an appropriate concentration of produced water is used, and a public risk assessment is performed.
